# 3D Printing—A Cutting Edge Technology for Treating Post-Infarction Patients

**DOI:** 10.3390/life11090910

**Published:** 2021-09-01

**Authors:** Daniel Cernica, Imre Benedek, Stefania Polexa, Cosmin Tolescu, Theodora Benedek

**Affiliations:** 1 Center of Advanced Research in Multimodal Cardiovascular Imaging, Cardio Med Medical Center, 540124 Targu Mures, Romania; daniel.cernica@umfst.ro (D.C.); imrebenedek@umfst.ro (I.B.); tolescu.cosmin@yahoo.com (C.T.); theodora.benedek@umfst.ro (T.B.); 2 Cardiology Department, University of Medicine, Pharmacy, Sciences and Technologies “George Emil Palade”, 540142 Targu Mures, Romania

**Keywords:** 3D printing, additive manufacturing, anatomic model, regenerative cardiology, interventional cardiology

## Abstract

The increasing complexity of cardiovascular interventions requires advanced peri-procedural imaging and tailored treatment. Three-dimensional printing technology represents one of the most significant advances in the field of cardiac imaging, interventional cardiology or cardiovascular surgery. Patient-specific models may provide substantial information on intervention planning in complex cardiovascular diseases, and volumetric medical imaging from CT or MRI can be translated into patient-specific 3D models using advanced post-processing applications. 3D printing and additive manufacturing have a great variety of clinical applications targeting anatomy, implants and devices, assisting optimal interventional treatment and post-interventional evaluation. Although the 3D printing technology still lacks scientific evidence, its benefits have been shown in structural heart diseases as well as for treatment of complex arrhythmias and corrective surgery interventions. Recent development has enabled transformation of conventional 3D printing into complex 3D functional living tissues contributing to regenerative medicine through engineered bionic materials such hydrogels, cell suspensions or matrix components. This review aims to present the most recent clinical applications of 3D printing in cardiovascular medicine, highlighting also the potential for future development of this revolutionary technology in the medical field.

## 1. 3D Printing in Cardiology—Introduction

Although three-dimensional printing has been commonly used in commercial fields in the last decade, the development of this new technology in medical field is still limited to several disciplines such as orthopedics, neurosurgery, maxillofacial surgery and cardiology [1,2]. In the cardiovascular field, besides research applications, 3D printing has been used for advanced visualization, improved diagnostic workflow, therapy guidance, simulation of minimally invasive and surgical procedures, and improvement of patient-physician communication [3,4,5,6,7,8,9].

The recent emergence of complex renderings of cardiac magnetic resonance (cMRI), cardiac computed tomography (CCT) and echo imagery have improved the visualization of pathologies, but lack in tactile experience and in three-dimensional complex visualization [10,11]. Conventional imaging techniques, such as CCT and cMRI, are mandatory to plan a complex intervention in patients with abnormal cardiac anatomy. However, they may provide limited spatial orientation in specific cases, giving that classic 3D renderings are displayed on a 2D screen. 3D printing technology can offer supplementary anatomic details to aid in pre-interventional planning, especially in cases characterized by complex 3D spatial relationships [12,13].

## 2. 3D Printing in Medical Applications—Basic Concepts and Technology

The development of a patient-specific prototype of 3D anatomical models starts with the acquisition of the clinical images from volumetric imaging data, converted then to a 3D digital format and finally to a physically printed model [14,15]. Basic imaging dataset may be obtained from cardiac MRI (cMRI), cardiac CT, transesophageal echocardiography (TEE), trans-thoracic echocardiography or conventional angiography [5,16]. These image datasets are exported in DICOM (Digital Imaging and Communication in Medicine) standard format. Contrast-enhanced CT images are preferable for 3D digital reconstruction because of their superior spatial resolution, thin slices (0.625 mm), wide field of view, multidimensional reconstruction capabilities and differentiation of soft tissue from bone or calcified structures. These images are particularly well suited for replicating cardiac chambers, large cardiac vessels, heart valves, and sub-valvular apparatus morphologies. As an alternative, cMRI provides good spatial and temporal resolution and enables noninvasive visualization of complex cardiac structures as well as quantitative assessment of flow features. In fact, cMRI has been widely used to assess and characterize congenital cardiac defects and to generate 3D-printed cardiovascular replicas with congenital defects. Three-dimensional TEE captures the motion of dynamic anatomical structures such as heart valve leaflets and sub-valvular apparatus elements; this allows the clinical team to select and assess the imaging time window of greatest interest [17,18,19,20]. Similarly, any 3D conventional angiographic modality (CT angiography, cMRI and rotational angiography) offers high quality imaging datasets to fabricate an endovascular model, for planning and simulation of endovascular intervention.

**Figure 1:**
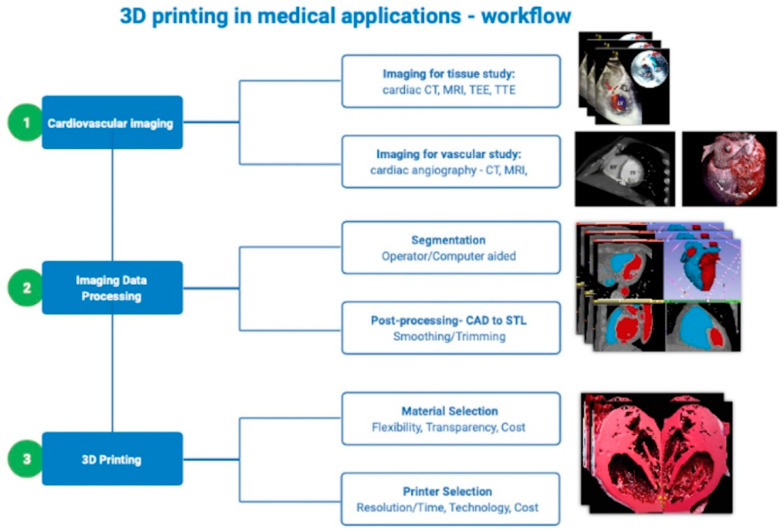
Cardiac 3D printing workflow.

3D printing workflow includes several steps, starting with acquisition of image data using of the previous mentioned imaging technologies (Figure 1). The next workflow step is image segmentation and post-processing to generate a typical digital format, namely STL or Standard Tessellation Language. Although several digital file formats are available, 3D printing model data is most commonly stored in STL format. For 3D printing technology, STL file format is the corresponding to DICOM format for conventional imaging. The STL file will be further post-processed using a computer-aided design (CAD) software system, which will complete the ultimate adjustments of the STL file before rapid prototyping. The final 3D model dataset is subsequently delivered to the 3D printer workstation to build the final physical models.

Usually, the unstable parts are printed with dedicated support platform to give desired position in space. In terms of time span of a fabricated 3D model depends mainly on the phantom size, structural complexity of the anatomical model and resolution/speed of the selected printer. A normal adult cardiac model may take 24 h to achieve the complete process. At the final phase, the 3D stereo-lithographic model often includes a subsidiary material for support purposes, which requires removal either by operator or by automated machine spreading special solutions to achieve a clean model. Once this stage is completed, and the extra-support material has been removed, the ultimate 3D anatomic model is exposed and ready to be used [2].

**Figure 2:**
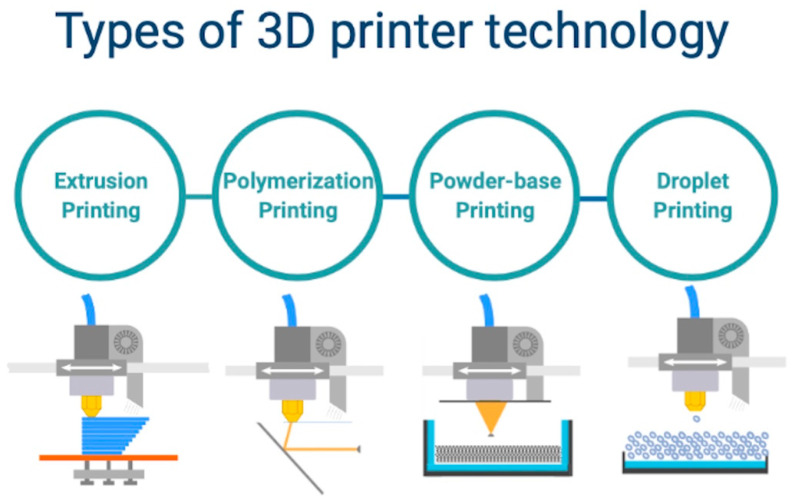
Types of 3D printer technology.

Printing technologies can be classified into four main categories (Figure 2):

- Printing by using the polymerization method which includes stereolithography and digital light processing; it consists of a laser beam directed to a selected resin material to cure layer by layer [21]; phantoms printed with this technique generally have predictable mechanical properties (isotropic); this method can be used to mimic soft and hard tissues or to fabricate complex anatomical parts, including cardiac valves; the major advantage of this technique consists in the possibility to print complex structures with refined surfaces; however, this method requires higher cost for the used materials and printing machines [22,23].
- Powder-based printing includes selective laser sintering or melting and consist of focal heating beam to fuse the selected powdered materials [24]; materials compatible with this method range from plastic powder to ceramics and metal alloys, which gives it a major advantage; nevertheless, it is considered the most expensive method due to increased costs related to the mechanical parts of the printing system and print materials [25].
- Printing by using the droplet method requires multi-jet modeling with wax or trough laser beam induced forward transfer, which is commonly used to deploy photo-curable resins into layers [26]; the technique usually uses photo-polymer bio-resins to fabricate scaffolds for tissue development; the advantage consists of an augmented printing precision regardless of the model complexity and dimensions; however, it requires an extended time frame for model prototyping [27];
- The extrusion printing method includes a fused deposition and direct ink writing modeling, by using a thermo-sensitive plastic or ink materials, which are delivered through a special printhead, layer by layer on a dedicated support area [28]; the use of thermoplastic ink or hydrogels facilitates the fabrication of anisotropic models with increased density, that are able to resist high loads and increased strain; despite having a lower precision, this method uses the lowest costs in terms of materials and printing machines [29].

## 3. Selection of Materials for 3D Printing

Material selection for 3D printing depends on the processing technique and the main field of activity in which the models are used. The variety of materials ranges from rigid to flexible, and also complex mixtures for combined properties.

### 3.1. Rigid Materials

Rigid materials are used mostly in pre-interventional planning models, which have been demonstrated to improve the spatial and structural understanding. The most commonly used printing materials are acrylonitrile butadiene styrene with fused filament modeling; this is considered as the most cost-effective material in the field of rapid prototyping. Moreover, the droplet method with thermoset materials has been used to fabricate appropriate devices for patient-specific models for simulation of flow resistances, with no model fractures, regardless of the fluid flow profiles. Metals such as titanium mostly used for printing implantable devices, due to its low weight, biocompatibility and sterilization adequacy [30].

### 3.2. Flexible Materials

By using flexible materials, printed models can attain various real-life haptic characteristics. This facilitates prototyping of 3D models with a wide range of flexibility and physical strength. For instance, a flexible material frequently used is thermoplastic polyurethane (TPU) which is an elastomeric resin, with a very high elasticity, flexibility and durability. This material frequently used in powder-based techniques can reproduce a wide variety of organs, tissues and vessels, thus allowing surgical and interventional simulations [31]. Similarly, the droplet-based process allows manufacture of flexible models, with TPU elastomers, which have a high UV resistance and resilience after deformation. Therefore, TPU models are suitable for incisions and suturing training. Another material with similar properties includes the flexible filament, which is suitable for 3D printing with the use of the extrusion process, with the advantage of being cost-efficient [32].

### 3.3. Printing with Multiple Materials

Multi-material compounds represent the future development of rapid prototyping models, that may be used to achieve realistic characteristics such as elastic and biological compatibilities. Three-dimensional printing by using fibers that appropriately regulate the mechanical performance of the physical model is currently under development [33].

## 4. Clinical Applications of 3D Printing in Cardiology

**Figure 3:**
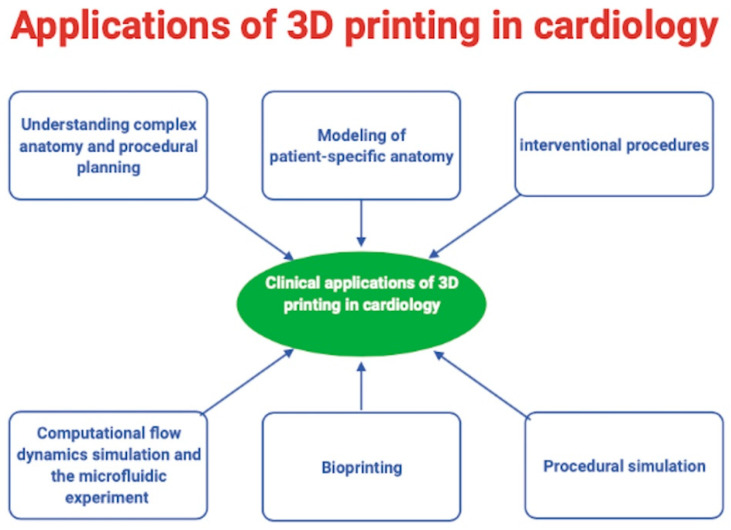
The main applications of 3D printing in cardiology.

The main applications of 3D printing in cardiology being experimented in present are illustrated in Figure 3.

## 5. 3D Printing to Understand Complex Anatomy and Procedural Planning

Stereolithography increases the stage of comprehensibility, especially in rare congenital heart disease or in post-myocardial mechanical complication, as each patient has specific cardiovascular anatomy and encompassing structures [17,34]. Congenital heart diseases imply a multitude of cardiovascular structures in a complex and unique pattern; Therefore, 3D-printed models have been shown to improve pre-procedural judgement and technical planning in these cases [35,36]. Printed phantoms have high resolution, with precise and smooth surfaces. It has been demonstrated by Valverde et al. that the pre-procedural approach was preserved after evaluation of the 3D model in 96.7% of the cases, while 96% of surgeons agreed that rapid prototyping models improved comprehensive status and technical planning [37]. Zhao et al. studied 25 patients with double outlet right ventricle and demonstrated that procedural times associated with cardiopulmonary bypass and cross-clamp were improved by pre-procedural 3D-printed model aid [38]. Olejník et al. studied rapid prototyping in tetralogy of Fallot with or without major arterio-pulmonary collateral arteries, showing that printed models of complex congenital heart defects facilitate pre-procedural decision and intraoperative orientation [39].

Schmauss et al. studied the role of surgery planning by rapid prototyping in patients with primary cardiac tumors, and demonstrated that 3D prototyping facilitates the diagnosis and therapeutic process by detailing the precise location and dissemination of the cardiac neoplasm into cardiac layers, especially when conventional imaging (CT, MRI or echocardiography) was unsatisfactory. They concluded that 3D printing enhanced both theoretic and intraoperative orientation for cardiac surgeons treating complex cardiovascular diseases [40,41,42]. Moreover, an experimental study conducted by Bateman and coworkers at the University of Minnesota’s Academic Health Center proved the importance of rapid prototyping technology in decision making in complex congenital heart disease [43]. In addition, they demonstrated the benefits of 3D printing engineering in a case of conjoined children. The curative surgical procedure was directed secondary to creation of 3D models of both hearts revealing a previously unnoticed cardiac connection [44].

## 6. 3D Printing for Procedural Simulation

The innovative potential of 3D printing includes facilitation of pre-procedural simulation for advanced intra-operative orientation and patient-specific tailored approach. Several experimental trials tried to assess whether rapid prototyping is applicable with simulation. Although the droplet printing process is expensive, it is capable to produce high precision 3D models. Additionally, it can use a multitude of polymer-based materials leading to fabricate models with variable density and flexibility to achieve real-like physical properties. Hazeveld et al. conducted a study to compare various 3D printing processes in terms of dimensional accuracy. They concluded that the highest dimensional precision method was the ink-jet technique [45]. For instance, Shiraishi et al. generated twelve 3D models by using both solid resin and flexible urethane materials, to simulate surgical and catheter intervention. The bio-models allowed better visual and tactile examination of anatomic specimen [46,47]. Motwani et coworkers described the utility of rapid prototyping models from CCT in several cases of aortic paravalvular leak. Giving a novel approach by transcatheter closure of paravalvular leak, 3D models allowed simulation of various treatment devices, with satisfactory outcomes [48]. Another application of modeling for procedural simulation was recently studied by Engelhardt and colleagues, who printed for the first time an integral mitral valve apparatus. Thus, cardiac surgeons could simulate various surgical techniques, such as annuloplasty, chordae implantation and leaflet-plasty. They concluded that 3D models were of superior quality and offered a high realism perspective by haptic interaction with surgical materials [49].

## 7. 3D Printing for Modeling of Patient-Specific Anatomy

A common complication of atrial fibrillation is represented by thrombosis of the left atrial appendage. According to international guidelines, percutaneous closure of the left atrial appendage is a treatment strategy adopted in selected patients, in order to prevent systemic embolization of such thrombus. Selection of the best dimensions and closure devices may be difficult in some cases with difficult anatomy. The powder-based printing method is widely utilized in producing bio-compatible implants. Considering the availability of a wide range of compatible materials, such as titanium alloy, zinc alloy, and cobalt-chrome alloy, and their mechanical characteristics and biocompatibility, this technique is the most promising in fabricating implants in cardiovascular surgery or percutaneous cardiovascular intervention field. This method is also the most appropriate to fabricate sterilizable devices [50]. In a recent study, Fan et al. emphasized the incremental outcome provided by association of TEE imaging and 3D printing technology in patients selected for left atrial appendage closure. They analyzed 72 patients with LAA occlusion device, comparing a group with TEE imaging-guided procedure with a group with 3D-printed guided procedure. The study results showed that the use of stereolithographic models improved the procedural outcome in terms of safety and efficacy, lowering the procedural duration, complications and MACE at three years follow up [51]. The innovative potential of fabricated 3D-printed models was validated in a similar study by Lazkani et al., who studied the role of rapid prototyping models in the surgical management of patients with ventricular septal defect following a myocardial infarction. They described the first case of a 3D-printed cardiac model from cardiac CT imaging used to guide a percutaneous closure of VSD, and reported that 3D printing technology provides the advantage to in-vitro assess the compatibility with multiple devices in terms of size and occluder type, at the same time improving the operator perspective and orientation among cardiac structures in fabricated model [52].

## 8. 3D Printing to Assist Interventional Procedures

Lodziński et al. described the role of 3D printing to assist complex electrophysiology procedures, in a case referred for ventricular tachycardia ablation in whom aberrant anatomic relationship between right ventricular outflow tract and right coronary artery was identified. A rapid prototyping model may be generated based on cardiac CT imaging, providing detailed information about anatomic relationship between cardiac structures, thus facilitating ablation of cardiac arrhythmia [53,54,55,56]. For instance, according to Rossi et al. work, for the rapid prototyping of the atria are usually used thermoplastic materials or rigid resin with extrusion process or powder-based process in order to obtain the most accurate anatomical model to merge with electrophysiology mapping system [57]. Yeazel et al. studied a novel approach in terms of percutaneous coronary stenting procedures, using 3D-printed scaffolds based on bio-resorbable materials and shape memory structure pattern. They described several advantages of 3D printing in interventional cardiology, such as tailoring patient-personalized production of devices or improving the management of stent migration or other complications [58,59]. Table 1 summarizes the most relevant studies published about the role of 3D printing technology for assisting cardiovascular interventions, together with their main results.

## 9. 3D Printing for Computational Flow Dynamics Simulation

The main use of computational flow dynamics (CFD) simulations is to better comprehend of the relationship between vessels, hemodynamics and development and progression of cardiovascular disease. Experiments for 3D printing and CFD simulation require the combined use of rapid prototyping models with a flow measurement system by MRI or particle image velocimetry [70]. Zelicourt et al. studied three-dimensional replication of a complex computational fluid dynamics system in a printed model for total cavo-pulmonary connection. The model was printed by using the stereolithographic technique with thermo-plastic resin, completed with a transparent acrylic paint. The study concluded that their model was appropriate for CFD dynamics simulations on vascular and cardiac structures, although the limitation was an increased model rigidity. [71]. Maragiannis and coworkers studied anatomical and functional characteristics of severe aortic stenosis using patient-specific models from cardiac-computed tomography. They used 3D printers with multi-material system to reproduce various structural lesions of the aortic valve. Thus, by combining conventional cardiac imaging, computed aided processing and 3D printing of patient-specific models could represent a novel technique to assess anatomy and functional characteristics of valvular heart diseases [72,73]. Functional post-interventional follow-up using rapid prototyping was also studied. According to Qian et al., paravalvular leak after trans catheter aortic valve replacement may be better assessed by using 3D models from cardiac CT imaging, and a novel index was described for prediction of paravalvular leak. This index, namely the annular bulge index, is based on metamaterials to mimic integral properties of a biological valve in a 3D environment [74,75].

## 10. 3D Printing for Bioprinting

A novel landmark in rapid prototyping technology concerns bioprinting or tissue engineering, which aims to provide a promising research opportunity. It consists of printing, layer by layer, a complex structure to mimic a real tissue structure. The most suitable technique is the droplet process with hydrogel materials. The laser printing technique has also been used in experimental studies, demonstrating a high spatial control of the cellular environment [76,77]. The main potential advantage of 3D bio-printed models concerns especially the field of regenerative medicine having also applicability in drug delivery or medical nanotechnology [18,78,79,80]. Table 2 summarizes the most relevant studies about 3D bioprinting and their applications in cardiology and their main outcomes.

Bio-fabrication of 3D models integrated with tissue specific cells that can be directly delivered on damaged tissue represents a unique potential solution for myocardial regeneration after a myocardial infarction. Molecular 3D bio-printing could represent the next generation in terms of personalized patient-specific medicine. This complex technology could facilitate interventions guided by specific biomarkers, in addition to tissue-based biomarkers. The novel paradigm of personalized medicine combines conventional anatomy imaging, functional assessing, physiology quantification, and also molecular diagnosis. Bio-printed models can determine specific molecular tagets for diagnosis and intervention, and molecular 3D printing may provide the boundary of future research, such as the development of imaging modalities with 3D printing technology, combined with genomics and molecular target labelling [81].

The most used systems in bioprinting are laser direct writing, inkjet extrusion, and cell electrospinning. Basara et al. produced a tissue engineering patch for patients with myocardial infarction, using a 3D-printed conductive titanium carbide integrated in a poly-etilen-glycol hydrogel [14,82]. Their results showed that bio-printed human-induced pluripotent stem cells derived cardiomyocytes synchronous beating, in parallel with an incremental expression of cardiac genes, thus creating a physiologically-like environment [77,83,84,85,86,87]. Similarly, Izadifar et al. studied a 3D-printed hybrid cardiac patch enriched with human coronary artery endothelial cells, using a combination of 3D-printed carbon nanotubes and collagen. At 10 days of in-vitro incubation, a network of nanofilaments was observed with a size up to 50 nm, with electrical and contractile improved activity. Moreover, stem cells presented a high level of proliferation and differentiation. Nowadays, the three-dimensional bioprinting represents a paramount in the scientific development. Though, it raises an ethical controversy about methods of rapid prototyping and cells or biomaterials manipulation. Specific approach and guidelines are needed to be designed in future [88,89,90].

**Table 2:** Applications of 3D bioprinting in cardiology.

First Author	Clinical Application	Supportive Material	Printing Method	Year	Country	Objective	Results
Gozde B. et al. [88]	Conductive composite in treatment of myocardial infarction	Titanium carbide mxene	Aerosol Jet Printing	2020	USA	To investigate cardiac patches as conductive myocardial tissue	Increase of MYH7, SERCA2, TNNT2 expression and conductive velocity
Izadifar M. et al. [89]	Intracoronary hybrid cardiac patch in ischemic heart disease	Methacrylated collagen	UV-integrated 3D-bioprinting-hydrogel	2017	Canada	To investigate hydrogel carboxyl functionalized carbon nanotubes	3D-bioprinted nanotubes presented significant cellular proliferation, migration and differentiation incubation time
Yeung E. et al. [91]	Regenerative patch in heart failure	Biomaterial-free	Monolayer bioprint on needle array	2019	USA	To investigate bioprinted patch in vivo scaffold free	Increased the average vessel counts patch group; the scar area in the cardiac patch group was significantly smaller
Gaetani R. et al. [92]	Role of epicardial tissue in myocardial infarction	Gelatin and hyaluronic acid matrix (hystem matrix)	Bioscaffolder-ink jet	2015	Netherlands	To evaluate the role of a 3D-printed patch composed of human cardiac-derived progenitor cells in a hyaluronic acid/gelatin matrix	Reduction in remodeling and preservation of cardiac performance and increase in cardiac and vascular differentiation at four weeks follow up
Jang J. et al. [93]	Treatment of ischemic heart disease	Decellularized extracellular matrix bioinks	Bio-ink printing	2016	Korea	To evaluate 3D pre-vascularized stem cell patch of cardiac remodeling and fibrosis, and promotional effects of cardiomyogenesis and neovascularization	Reduction of cardiac remodeling and fibrosis by modulating inflammation, apoptosis, and cardiac metabolism; increasing of neovascularization
Gao L. et al. [94]	Treatment of ischemic heart disease	Photoactive gelatin polymer	Multiphoton-excited photochemistry printing	2018	USA	To evaluate bioprinted scaffold with cardiomyocytes, smooth muscle cells, and endothelial cells in a murine model of myocardial infarction	The scaffold promoted cell viability and electromechanical coupling in vitro, high levels of cell engraftment, as well as significant improvements in cardiac function, infarct size, apoptosis, vascularity, and cell proliferation in a murine MI model
Wang Z. et al. [95]	Heart failure treatment	Fibrin-based composite hydrogel	Bioink hydrogel	2018	USA	Develop a functional cardiac tissue in heart failure	Bioprinted cardiac tissue is not structural and functional like native tissue after printing, instead the tissues are matured after three to four weeks in culture
Park S. et al. [96]	Treating coronary thrombosis	Polycaprolactone mixed with poly-lactide-co-glycolide and polyethylene glycol	Rotational 3D printing	2014	Korea	To evaluate feasibility of 3D-printed biocompatible and biodegradable stent	It showed a reduction of neointimal hyperplasia, the inflammatory cells and fibrin infiltrated around the struts

## 11. Future Directions

Three-dimensional printing has been introduced in clinical practice, not only for research purposes. This method has the potential to trigger a paradigm shift towards a personalized approach in diagnostics and therapeutic management of various medical conditions. Three-dimensional models may also be used in a variety of cardiovascular applications, such as customizing tailored-patient devices, organ tissues or producing complex drugs delivery systems on-demand [97]. The most promising application in this field is the bioprinting of complex tissues and viable implants. The future steps could be made towards stem cells manipulation for growing specific tissues, that can be used as replacement or corrective tissues [98]. In situ three-dimensional printing is another expected advance, which consists of fabricating specific tissues in real time during a surgical intervention [77].

## 12. Conclusions

Three-dimensional printing has a great potential to revolutionize personalized medicine. This technology may be used for advanced diagnostic and treatment applications in various medical applications, such as peri-operative planning for complex congenital heart diseases, guidance of interventional procedures or procedural simulation. Recent experimental studies are directed to emerging applications of 3D printing, including bio-printing and molecular printing, which will allow a better approach of various cardiac pathologies. However, in the long term perspective, more experimental studies are needed to generate solid evidence regarding the benefits of this novel technology, as well as its feasibility in clinical practice.

## Figures and Tables

**Table 1:** 3D printing applications in cardiac interventions.

First Author	Clinical Application	Imaging Modality	3D-Printed Material	Printing Method	Year	Country	Objective	Results
Lazkani M. et al. [52]	Postinfarct VSD treatment	Computed tomography Angiography	Gypsum-cyanoacrylate	Stereolithography	2015	USA	To evaluate the role of 3D-printed occluder in guided percutaneous treatment of complex PIVSD	3D printing model guide size and choice of septal occluder with no residual shunt
Modi B. et al. [60]	Assess Coronary Artery Disease	Coronary angiogram	Photopolymer	Ink Jet	2018	United Kingdom	Three-dimensional-printing to characterize serial stenosis interplay in vitro	Generated and tested a mathematical equation to improve estimation of the true physiological impact of each stenosis, using measurements from a routine pressure-wire study
Oliveira-Santos M. et al. [61]	Simulation percutaneous coronary intervention	Coronary angiogram	Hybrid flexible filled with fluid	Stereolithography	2018	Portugal	Patient-specific simulation of percutaneous coronary intervention	Patient-specific simulation is feasible to guide the treatment strategy of complex coronary artery disease (e.g., treating a critical ostial Cx stenosis)
Wang H. et al. [62]	Optimized stent implantation strategy	Coronary angiogram	Polydimethylsiloxane	Wax Jet	2015	China	Optimized stent position in microfluidic settings through 3D printing technology	Microfluidic model has demonstrated to be a feasible and novel approach for hemodynamic study of coronary disease
Velasco M. et al. [63]	Diagnosis and interventional planning of coronary artery fistulae	Computed tomography and cardiac magnetic resonance	Polyjet photopolymer	Polyjet	2017	United Kingdom	To evaluate the 3D-printed models contribution to the diagnosis and interventional planning of coronary artery fistulae	Improvement in selection of the correct equipment and in reduction of procedure times
Sedaghat A. et al. [64]	Percutaneous treatment of coronary artery aneurysm	Computed tomography Angiography	Silicone	Stereolithography	2018	Germany	To investigate a multimodal preprocedural planning in percutaneous coronary aneurysm treatment	Confirm the benefit of ex vivo 3D-printed model in interventional treatment of an extensive coronary aneurysm
Mohamed E. et al. [65]	Percutaneous treatment of left ventricle pseudoaneurysm	Computed tomography Angiography	Clear resin	Stereolithography	2019	USA	To investigate the role of 3D model in complications of myocardial infarction	Improve the accuracy to cannulate the aneurysm, select the appropriate sheath/catheter shape and determine the most suitable occluder device type and size
Bompotis G. et al. [66]	Transcatheter aortic valve implantation	Computed tomography aortography	Clear resin	Stereolithography	2019	Greece	To investigate the role of 3D model in transcatheter valve implantation	It was demonstrated to be a significant tool for the optimal sizing and positioning of the transcatheter implantation of the bioprosthetic valves and the reduction of the para- valvular leak
Baribeau Y. et al. [67]	Complex procedures on patient-specific models	Transesophageal Echocardiographic	Clear resin	Stereolithography	2019	USA	Procedural simulation that use a pulsatile left-sided heart printed model	Provided a better understanding of the anatomy and assessing the effect of interventions
Iriarta X. et al. [68]	Percutaneous appendage closure	Computed tomography angiography	Rubber-like material	Stereolithography	2018	France	To study the role of cardiac three-dimensional printing in percutaneous appendage closure	3D model allow testing of several designs of prosthesis to better choose the optimal shape and size of the patient-specific prothesis to prevent procedural complications
Lodziński P. et al. [69]	Ventricular tachycardia ablation	Computed tomography angiography	Gypsum-cyanoacrylate	Stereolithography	2017	Poland	To investigate the role of 3D model in complex tachycardia treatment	Improve spatial orientation in patients with complex anatomy and provide a novel technology of mapping in ablation procedures

## Data Availability

Not applicable.
